# Biodegradation of PLA-PHBV Blend Films as Affected by the Incorporation of Different Phenolic Acids

**DOI:** 10.3390/foods11020243

**Published:** 2022-01-17

**Authors:** Eva Hernández-García, Maria Vargas, Amparo Chiralt, Chelo González-Martínez

**Affiliations:** Instituto Universitario de Ingeniería de Alimentos para el Desarrollo, Universitat Politècnica de València, 46008 Valencia, Spain; evherga1@upvnet.upv.es (E.H.-G.); dchiralt@tal.upv.es (A.C.); cgonza@tal.upv.es (C.G.-M.)

**Keywords:** antimicrobial, ferulic, *p*-coumaric, protocatechuic, disintegration, thermogravimetric analysis, biodegradation

## Abstract

Films based on a 75:25 polylactic acid (PLA) and Poly(3-hydroxybutyrate-co-3-hydroxyvalerate) (PHBV) blend, containing 2% (*w*/*w*) of different phenolic acids (ferulic, *p*-coumaric or protocatechuic acid), and plasticised with 15 wt. % polyethylene glycol (PEG 1000), were obtained by melt blending and compression moulding. The disintegration and biodegradation of the film under thermophilic composting conditions was studied throughout 35 and 45 days, respectively, in order to analyse the effect of the incorporation of the antimicrobial phenolic acids into the films. Sample mass loss, thermo-degradation behaviour and visual appearance were analysed at different times of the composting period. No effect of phenolic acids was observed on the film disintegration pattern, and the films were completely disintegrated at the end of the composting period. The biodegradation analysis through the CO_2_ measurements revealed that PLA-PHBV blend films without phenolic acids, and with ferulic acid, completely biodegraded after 20 composting days, while *p*-coumaric and protocatechuic slightly retarded full biodegradation (21 and 26 days, respectively). Phenolic acids mainly extended the induction period, especially protocatechuic acid. PLA-PHBV blend films with potential antimicrobial activity could be used to preserve fresh foodstuff susceptible to microbial spoilage, with their biodegradation under composting conditions being ensured.

## 1. Introduction

Biodegradable polymers and their compounds have a wide range of applications and are in high demand since they can be good alternative means of reducing the global environmental impact caused by non-biodegradable polymers and the concerns derived from their use [1]. It has been shown that this environmental impact has led to undesirable consequences on marine fauna, the climate, human health and even the economy. Additionally, the benefits of these biodegradable materials in terms of their renewable origin, safety, low cost and potential role for extending the shelf life and maintaining the quality of many food products have to be taken into account [2].

The global production of bioplastics was 2.11 million tonnes in 2020, and the forecast is for 2.87 million tonnes by 2025. Biodegradable plastics such as polylactide (PLA), polyhydroxyalkanoates, (PHAs), starch blends and others) represent 60% of the capacity (more than 1.2 million tonnes) of global bioplastics’ production. This production of biodegradable plastics is expected to increase to 1.8 million tonnes in 2025. Of the 60% of biodegradable plastics, 18.7% correspond to PLAs and 1.7% to PHAs [3].

PLA is a biodegradable polyester that is obtained from lactic acid during the fermentation of renewable crops—it is readily available and low in cost. It is characterised by its rigidity, transparency, processability and biocompatibility [4], and can be degraded by abiotic and biotic processes and even by a great variety of microorganisms [5]. Due to its low resistance to oxygen permeation and its brittleness [6], recent studies have focused on improving its functional properties, i.e., by combining PLA with other plasticisers and polyesters, such as PHBV [7,8,9,10]. Moreover, PHBV is one of the most widely used PHAs due to its lower degree of rigidity, compatibility and biodegradability [11].

In order to obtain active biodegradable films, active compounds are usually incorporated so as to confer antimicrobial and antioxidant properties on the final blend films [2,7,8,12]. Of the active compounds, phenolic acids, such as ferulic acid, *p*-coumaric and protocatechuic acids [13,14,15,16,17,18], have demonstrated significant antimicrobial and antioxidant properties while exhibiting a milder sensory impact than other natural compounds extracted from plants, such as essential oils.

Composting and recycling are the most common processes for the recovery of plastics. Composting is a subset of biodegradation [19] in which organic matter is transformed into CO_2_ and soil-like material (humus) by the activity of different microorganisms under aerobic conditions [20]. Changes in the formulation of bioplastics, including the incorporation of filler compounds, blends and active compounds such as antimicrobials, among others, could promote changes in their biodegradation pattern in different environments; however, these potential changes are a largely unexplored frontier [19]. Few studies can be found about the influence of the incorporation of antimicrobial compounds on the biodegradation behaviour of films under composting conditions. In some of these studies [21,22], no changes in the film’s biodegradation behaviour were found, such as was observed when sodium propionate [21] or silicon oxide nanoparticles [22] were incorporated as antimicrobials into starch/PVA blends. Other authors showed that the effect of the antimicrobials on the biodegradation profiles of the films depended on the type and concentration of the antimicrobial compound [23]. Thus, these changes were greater when using non-volatile substances with strong antimicrobial power, such as silver species, which slowed down the degradation rate and reduced the degradation extent, suggesting a partial alteration of the compost inoculum. Other authors [9,24] also showed that the presence of catechin [9] or silver nanoparticles [24] in PHBV-based films promoted a remarkable delay in the biodegradation process. The possible changes in the biodegradation pattern of the films when incorporating antimicrobials depended on their release kinetics into the medium, the sensitivity of the different microorganisms responsible for the degradative process and the dose of active compounds in the films [23].

The aim of the present study was to analyse the effect that the incorporation of ferulic, *p*-coumaric and protocatechuic acids with antimicrobial activity into PEG plasticised PLA/PHBV (75:25) blend films had on their disintegration and biodegradation behaviour under laboratory composting conditions.

## 2. Materials and Methods

### 2.1. Materials

Amorphous polylactide (PLA, 4060 D) with a density of 1.24 g/cm^3^ and average molecular weight of 106,226 D (40% low molecular weight fraction (275 D), as reported by [25]) was purchased from Natureworks (Minnetonka, MN, USA). Poly(3-hydroxybutyrate-co-3-hydroxyvalerate) (PHBV, ENMAT Y1000P) with 3% hydroxyvalerate was supplied by Helian Polymers B.V. (Belfeld, Holland). Poly(ethylene glycol) with a molecular weight of 1000 Da (PEG1000) and ferulic, *p*-coumaric and protocatechuic acids were purchased from Sigma-Aldrich (Madrid, Spain). Phosphorus pentoxide (P_2_O_5_) and magnesium nitrate-6-hydrate (Mg(NO_3_)_2_) were supplied by Panreac Química, S.A. (Castellar del Vallès, Barcelona, Spain).

For the biodegradation and disintegration studies, ripe compost (no more than 3 months old) from a local solid waste treatment plant (Valencia, Spain) was used. Vermiculite (Leroy Merlin Valencia, Spain) and cellulose microcrystalline (CMC) (Sigma Aldrich, Madrid, Spain) were used for the biodegradation test. Other components used in the disintegration test were refined corn germ oil (Koipe, Córdoba, Spain), corn starch (Roquette Laisa, Benifaio, Valencia, Spain), sucrose (Azucarera Ebro, Madrid, Spain), urea (Urea Prill 46%, Antonio Tarazona S.L., Silla, ValenciaSpain), rabbit food (Super Feed S.L, Madrid, Spain) and sawdust (Productos de limpieza Adrián, Valencia, Spain).

### 2.2. Obtaining Active Blend Films

PLA-PHBV blend monolayers were obtained by melt blending and compression moulding in a polymer ratio of 75:25, using PEG1000 (15 g/100 g polymers) as a plasticiser, according to a previous study [8], incorporated with 2% (*w*/*w* with respect to the film mass) antibacterial compound (ferulic, *p*-coumaric or protocatechuic acids). These phenolic acids were chosen on the basis of their previously proven antimicrobial activity [13,26]. The melt blending process was carried out in an internal mixer (HAAKE^TM^ PolyLab^TM^ QC, Thermo Fisher Scientific, Dreieich Germany) at 170 °C and 50 rpm, for 12 min. Four grams of dried pellets were placed onto Teflon sheets and preheated at 200 °C for 5 min in a hot-plate press (Model LP20, Labtech Engineering, Samut Prakan, Thailand). The films were obtained by compressing at 200 °C for 4 min at 100 bars, followed by a final cooling cycle for 3 min until the temperature reached about 70 °C [8]. The films were then conditioned at 25 °C and 53% RH.

### 2.3. Film Characterisation

#### 2.3.1. Moisture Content, Thickness, Elementary Composition and Visual Appearance

The moisture content was determined in previously conditioned films in triplicate. To this end, four samples per formulation were dried in a vacuum oven (J.P. Selecta, S.A. Barcelona, Spain) at 60 °C for 24 h and, subsequently, the samples were placed into a desiccator with P_2_O_5_ at 25 °C for 2 weeks, until they reached a constant weight [10]. The film thickness was measured at 5 different points using a Palmer digital micrometre (Comecta, Barcelona, Spain).

In order to determine the C composition of the samples, an elemental analysis was performed by means of a Euro EA3000 analyser (EUROVECTOR, Milan, Italy). The analyses were carried out in triplicate.

The morphological changes that the samples underwent throughout the disintegration experiments were recorded by means of photographs taken at different times of the process. For this purpose, specific samples extracted from the reactors were dried in a vacuum oven at 40 °C for a week and the pictures were taken using a digital camera (EOS 5D Mark II, Canon, Tokyo, Japan).

#### 2.3.2. Thermogravimetric Analysis

The thermal analysis of film samples was carried out in triplicate using the thermogravimetric analyser (TGA 1 Star^℮^ System analyser, Mettler-Toledo, Switzerland) at 0, 14, 21 and 35 disintegration days. Previously, the samples were conditioned in desiccators with P_2_O _5_. For the analysis, the samples were heated from 25 to 600 °C at 10 °C/min under a nitrogen atmosphere (10 mL/min). From the curves obtained in each test, the derived curves (DTGA) as a function of temperature and the corresponding values of T_max_ (temperature of maximum degradation rate) were obtained using the STAR^e^ Evaluation Software Version 16.20.12030 (Mettler-Toledo, Greifensee, Switzerland).

### 2.4. Compost and Synthetic Solid Residue (SSR)

For the disintegration and biodegradation tests, ripe compost supplied from a local solid waste treatment plant was used, which was previously prepared by removing any inert pieces and then sieved. Its pH was assessed by mixing one part of compost to five parts de-ionised water and measured immediately. A synthetic solid residue (SSR) was prepared for the disintegration test by manually mixing ripe compost, corn germ oil, corn starch, sucrose, urea, rabbit food and sawdust, according to ISO 20200 [27].

For both tests, the water content of the ripe compost and of the SSR was adjusted to 55% (*w*/*w*) throughout the experimental period by incorporating de-ionised water and gently stirring. The characterisation of dry (DS) and volatile (VS) solids was carried out in triplicate at the beginning and at the end of the composting process, following the ISO 20200:2004 International Standard [27]. DS was obtained by drying the sample in a convection oven at 105 °C until constant weight (Equation (1)) and VS was determined by calcining the samples previously dried in a muffle (Selecta, Barcelona, Spain) at 550 °C until constant weight (Equation (2)). The ripe compost used for the biodegradation test was also characterised in terms of DS and VS at the initial time.

(1) $$\mathrm{DS}\left(\%\right)=\frac{W_{d}^{105}}{W_{w}^{i}}\times 100$$

(2) $$\mathrm{VS}\left(\%\right)=\frac{W_{d}^{105}-W_{d}^{550}}{W_{d}^{105}}\times 100$$

where $W_{w}^{i}$ is the initial weight of the sample, $W_{d}^{105}$ is its weight after drying at 105 °C and $W_{d}^{550}$ is the weight of the ashes after the treatment at 550 °C.

### 2.5. Disintegration Test

The film disintegration test was performed on a laboratory scale, following an adapted method based on the current ISO 20200:2004 International Standard [27].

Film samples (cut into 25 × 25 mm squares) included in mesh bags (1 × 1 mm mesh size) were weighed using an analytical balance (±0.00001 g) and placed into reactors (less than 10 g per reactor) with 1 kg of SSR. Three reactors were prepared per film formulation, which were stored in an oven (Selecta, J.P. Selecta S.A., Barcelona, Spain) at 58 ± 2 °C, to ensure controlled thermophilic conditions, for thirty-five days. A 5 mm diameter hole was made on each narrow side of the reactor to allow gas exchange between the atmospheres on the inside and the outside. At the initial time and throughout the 35 days of testing, the reactors were weighed, and if necessary, de-ionised water was added to restore the initial mass, as specified by the ISO 20200:2004 International Standard [27]. One of the samples with around 5 g (cut into 25 × 25 mm squares) was used to control the final time sample weight loss, according to the standard guidelines, and the other mesh bags, each with one sample square, were extracted from the reactor at different control times in order to carry out both the TGA and the visual morphological analysis. Prior to these analyses, the mesh bags containing samples were gently cleaned with a soft brush to eliminate the adhered compost residues.

The disintegration percentage after the 35-day composting period was determined according to Equation (3), on the basis of the mass loss of the film:

(3) $$D_{35}\left(\%\right)=\frac{m_{0}-m_{35}}{m_{0}}\times 100$$

where m_0_ is the sample dry mass at the start of the test and m_35_ is the dry mass of the final disintegrated samples after 35 composting days.

### 2.6. Biodegradation Test

The adapted ISO 14855:1-2012 International Standard [28] method was used for the analysis of the aerobic biodegradability of films under controlled composting conditions, by measuring the amount of generated CO_2_. Prior to the test, the C, N and H contents of the different samples were determined. In this test, ripe compost was mixed with vermiculite (compost/vermiculite ratio: 3:1) to prevent the compaction of the compost and to ensure good oxygen access. For this analysis, reactors (glass jars of 2 L) containing two polypropylene flasks (60 mL) were used: one of them contained 3 g of dry compost mixed with 1 g of vermiculite and a sample quantity (previously cut into 2 mm^2^ squares) equivalent to 50 mg of carbon, while the other flask contained de-ionised water to ensure 100% relative humidity. The bioreactors were closed and incubated for 45 days at 58 ± 2 °C. A blank reactor containing only compost and another containing cellulose microcrystalline (CMC) as a reference sample [29], mixed with the compost, were also prepared.

The percentage of CO_2_ generated inside the reactors was measured in triplicate using a CO_2_ analyser throughout the biodegradation process. The theoretical amount of CO_2_ that could be generated from the sample (CO_2_^Th^S) was estimated from its carbon content by applying the following equation (Equation (4)):

(4) $${\mathrm{CO}}_{2}^{\mathrm{Th}}S={\mathrm{DW}}_{s}\times C_{s}\times \frac{{\mathrm{Mw}}_{\mathrm{CO}2}}{{\mathrm{Mw}}_{C}}$$

where DWs is the dry weight of the sample (g), $C_{s}$ is the proportion of carbon in the dry sample, as determined by elementary analysis (%), and ${\mathrm{Mwco}}_{2}{\;\mathrm{and}\;\mathrm{Mw}}_{C}$ are the molecular weights of CO_2_ and of C, respectively.

The percentage of biodegradation was calculated using the following equation (Equation (5)) [29], assuming that all sample carbon was converted into CO_2_:

(5) $$B\left(\%\right)=\frac{\sum {\mathrm{CO}}_{2S}-\sum {\mathrm{CO}}_{2B}}{{\mathrm{CO}}_{2}^{\mathrm{Th}}S}\times 100$$

where $\sum {\mathrm{CO}}_{2S}$ is the accumulative amounts of CO_2_ produced in the sample bioreactor and $\sum {\mathrm{CO}}_{2B}$ is the accumulative amounts of CO_2_ produced in the blank bioreactor.

To describe the kinetics of the process, the experimental data obtained from the biodegradation test were modelled using the Hill equation (Equation (6)):

(6) $$B\%={B\%}_{\max}\times \frac{t^{n}}{k^{n}+t^{n}}$$

where B%_max_ is the percentage of biodegradation at infinite time (%), t is the time, k is the time at which 50% of the maximum biodegradation occurred (0.5% B_max_) and n is the curve radius of the sigmoid function.

### 2.7. Statistical Analysis

Statgraphics Centurion XVII-X64 software (Manugistics Corp., Rockville, MD, USA) was used to perform the statistical analyses of the results by means of an analysis of variance (ANOVA). Fisher’s least significant difference (LSD) procedure was used at the 95% confidence level.

## 3. Results and Discussion

### 3.1. Moisture Content, Thickness and Elementary Composition

Table 1 shows the initial moisture content, thickness and carbon content of PLA-PHBV control films with and without phenolic acids. The moisture content of all the films was very low, coinciding with the hydrophobic nature of the polymeric matrix. The incorporation of the phenolic acids slightly reduced (*p* < 0.05) the moisture content of the films, with the sample containing protocatechuic acid exhibiting the lowest moisture content.

Film thickness ranged between 140 and 150 µm and the incorporation of phenolic acids had a significant effect on the thickness of the films. Ferulic acid led to an increase in the film thickness, whereas protocatechuic acid led to a significant decrease. This could be associated with the different interchain forces established between polymers and the antimicrobial agents, depending on the phenol molecular structure, which could affect the flowability of the material during the thermo-compression process and thus, the final film thickness. Ferulic and *p*-coumaric acids are hydroxycinnamic acids with a para OH group, but ferulic acid also contains a meta methoxy group in the molecule. This could promote higher interchain distance when phenol–polymer hydrogen bonds are formed. In contrast, protocatechuic is a hydroxybenzoic acid with two hydroxyls in meta and para positions, which could promote lower interchain distances by the hydrogen bonding.

The carbon content of the samples (Table 1) was about 52% for all the samples, none of which differed significantly, as expected from their very similar composition (only 2 wt.% of the different phenolic acids in the films).

### 3.2. Compost Characteristics

The ripe compost used as inoculum in both tests showed an initial organic matter content (VS) of 93.4% ± 1.2%, expressed as volatile solids with respect to dried solids. The content of total dry solids (DS) was 85% ± 1% and pH = 7.8, measured according to ISO 14855 [28]. At the end of the disintegration test, the content of organic matter in the compost changed and the values are shown in Table 2 for the different reactors without films and with the different film samples. All the values decreased with respect to the initial value by more than 40%, in line with the conversion of the organic matter into CO_2_ brought about by the microflora of the compost throughout time. The percentage of organic matter reduction was higher in reactors containing films, which suggests that inoculum could be stimulated by the film substrates. The standard method establishes a reduction in the volatile content in the sample after the composting period (R values in Table 2) of over 30%, as well as a standard deviation of the disintegration values of the samples (D_35_ (%), in Table 2) of less than 10 units. Likewise, the appearance of the composting material changed over time, from an initial yellow to a brownish colour after 10 days of composting, according to what is described in the ISO 20200 standard [27]. Therefore, the test could be validated.

### 3.3. Disintegration Test

**Figure 1:**
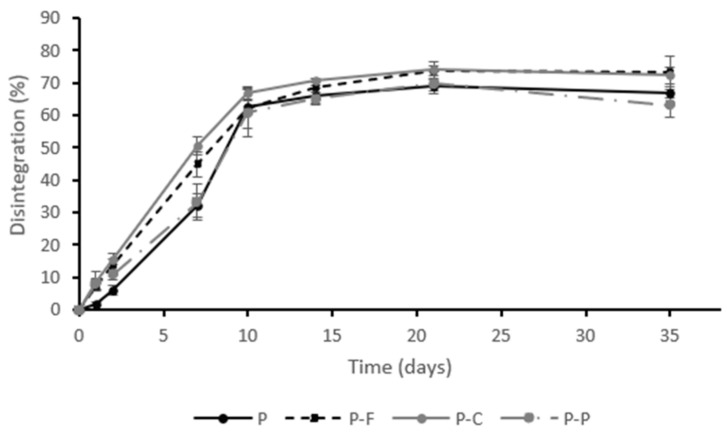
Development of sample disintegration as a function of time for the different films. PLA-PHBV blend films (P) and PLA-PHBV blend films containing ferulic acid (P-F), *p*-coumaric acid (P-C) and protocatechuic acid (P-P). Mean values and standard deviations.

Figure 1 shows the development of the disintegration (mass loss) of the different films as a function of time. As can be observed, all the films showed similar disintegration patterns. During the first 10 days, the mass losses occurred very fast in every case, reaching %D values of around 63–67%. This disintegration rate was greater than those found by other authors for PLA-PHBV blends [30,31], probably due to the presence of a plasticiser (PEG1000) and to the amorphous nature of PLA, which both lead to an acceleration of the process. The disintegration rate for the PLA-PHB blend and the PLA-PHB blend with limonene in the first 10 days was less than 10% [30], while for the PLA-PHBV blend it was 13.2% after 30 days [31].

From 10 days on, the disintegration rate slowed down, reaching a plateau after around 30 days, and the sample weight losses reached values of close to 90% (Table 2). In general, the incorporation of antimicrobial agents did not induce significant changes (*p* > 0.05) in the disintegration behaviour of P films.

**Figure 2:**
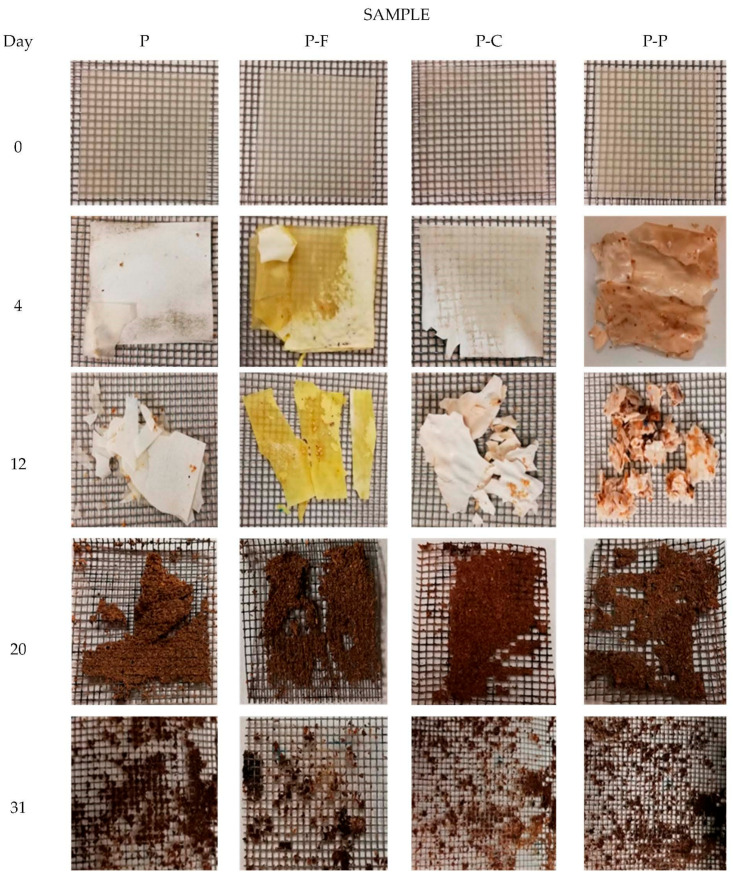
Photographs of the film samples after different composting times. PLA-PHBV blend films (P) and PLA-PHBV blend films containing ferulic acid (P-F), *p*-coumaric acid (P-C) and protocatechuic acid (P-P).

Photographs after different composting times (Figure 2) show the morphological changes in film samples associated with their disintegration. As can be observed, considerable erosion and fractures were found in the films after 4 composting days, and after 12 days, the samples appeared broken into small pieces, especially the P-P films. After 20 composting days, the film’s appearance in the mesh was no longer distinguishable from compost due to the fact that the aggregates were brown, making the weighing process more difficult. Afterwards, the mass particles disaggregated, becoming smaller than the mesh size, leading to a very small residue, coinciding with the complete disintegration of the films. From these observations, it can be concluded that the film disintegrated completely before 30 days, although small fragments were adhered to the mesh, contributing to the values of residual mass shown in Figure 1.

The colour of the formulations incorporating ferulic and protocatechuic acids became more yellowish and brownish throughout the composting period, respectively, due to the promotion of the oxidation of these phenolic compounds at the beginning of the disintegration process.

### 3.4. Thermogravimetric Analysis

**Figure 3:**
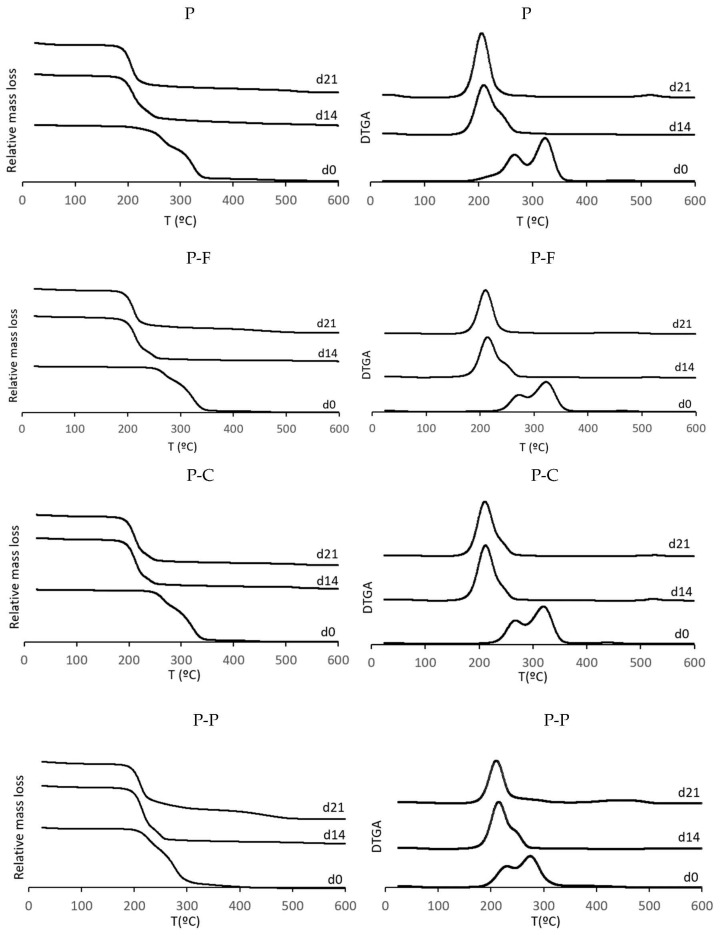
TGA and DGTA curves of PLA-PHBV blend films (P) and PLA-PHBV blend films containing ferulic acid (P-F), *p*-coumaric acid (P-C) and protocatechuic acid (P-P), after different composting times (0, 14 and 21 days).

The thermal degradation behaviour of PLA-PHBV blend films and of those containing phenolic acids was studied by TGA at different times of the disintegration period to observe the changes in the polymer structure. The TGA curves and their first derivatives are depicted in Figure 3. The onset (T_0_) temperature of degradation and the temperature at which the maximum degradation rate (T_peak_) occurred for the different degradation steps of the films at different times of the disintegration test are also summarised in Table 3.

As can be observed, all the films exhibited a two-step mass loss before the composting process (*t* = 0). These steps can be attributed to the separate thermo-degradation of PLA and PHBV, in line with what has previously been reported [8], which is a typical pattern when blending immiscible polymers with different thermal stabilities. Thus, PLA, which was more thermostable than PHBV [31], showed a maximum degradation rate at 320 °C, at which temperature the cleavage of bonds on the backbone to form cyclic oligomers, lactide and carbon monoxide as products occurs. As concerns the PHBV, the maximum degradation rate occurred at 268 °C, corresponding to a random intramolecular chain scission by cis-elimination, which leads to a remarkable molecular weight reduction and, to a lesser extent, to an intermolecular chain trans-esterification [32]. Similar thermo-degradation patterns were obtained by other authors for plasticised PHBV [33]. In general, the incorporation of ferulic acid and *p*-coumaric acid increased the onset temperature (*p* < 0.05) of the thermal degradation process, whereas the incorporation of protocatechuic acid caused a significant decrease in the thermal stability of the film, in agreement with that previously observed [34]. Phenolic acids usually promote a crosslinking effect between polyester chains, which could explain the observed changes in the onset of the thermal degradation of polymers [35]. Additionally, as suggested by other authors, the protective effect of the antioxidant agents against polymer thermal degradation at the first heating stages has to be considered [36]. In contrast, protocatechuic acid is found to inhibit PHBV crystallisation, thus favouring its thermo-degradation at lower temperatures [35]. Likewise, the phenolic acids affected the peak temperature of the polymer thermal degradation, in the same sense as that observed for the onset temperature.

As shown in Figure 3, the effect of the composting time was noticeable for the different degradation steps. Degradation peaks shift towards lower degradation temperatures throughout the first 21 composting days, and completely disappeared in samples composted for longer times. This was consistent with the remarkable reduction in the molecular weight of both polymers throughout this period. Thus, both the amorphous-PLA fraction and the semicrystalline PHBV fraction were partially hydrolysed, thus reducing their thermal stability. After 21 composting days, only one thermo-degradation peak was observed, which could indicate that amorphous PLA was completely disintegrated, and only the partially degraded crystalline PHBV remained, although both partially degraded polymers could be present with similar thermo-degradation patterns. After 35 days, the characteristic peaks of both PLA and PHBV completely disappeared as a consequence of the full degradation of the material. The residual components were more thermostable, exhibiting higher degradation temperatures (450 and 550 °C), as was observed in other studies [30,37].

Table 3 also shows the residual mass at 600 °C for each sample, which exhibited very low pyrolysis residual mass. This mass increased (*p* < 0.05) throughout the composting time, regardless of the incorporation of the phenolic acids. This was coherent with the increase in the mass ratio of the mineral content in the partially degraded polymeric film, as previously observed by other authors [37].

### 3.5. Biodegradation Kinetics under Laboratory-Scale Composting Conditions

Biodegradation is promoted by abiotic (surface erosion, temperature, humidity, pH, etc.) and biotic factors, where the microorganisms present in the environment surrounding the material consume oxygen under aerobic conditions and release enzymes, which allow the complete or partial decomposition of the material into carbon dioxide, water, inorganic compounds and biomass [38].

The biodegradation behaviour of the films in a composting environment was analysed by submitting the samples to laboratory-scale composting conditions for more than 40 days, using a constant temperature of 58 °C, following the ISO 14855 standard [28]. The theoretical maximum quantity of carbon dioxide that can be produced by the total biodegradation of the samples was calculated from their carbon content. The biodegradation profile of cellulose microcrystalline (CMC) was also analysed to be used as a reference.

**Figure 4:**
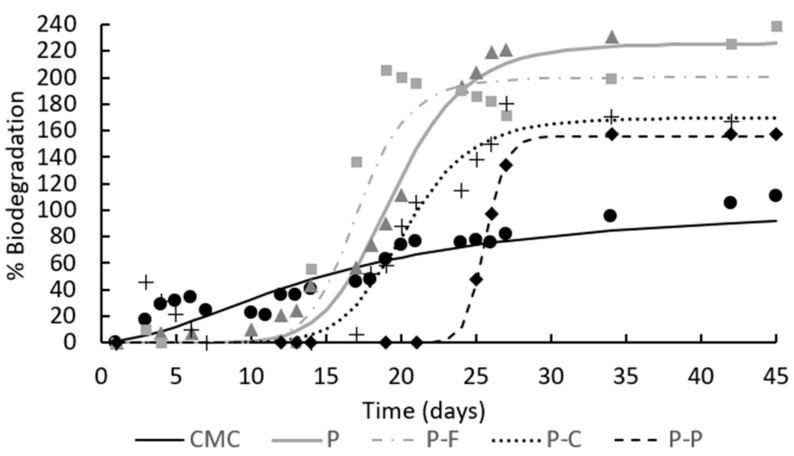
Biodegradation kinetics of CMC (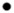) and PLA-PHBV films P (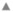), PLA-PHBV films containing ferulic acid P-F (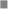), *p*-coumaric acid P-C (**+**) and protocatechuic acid P-P (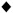) throughout the composting time. Experimental data (symbols) and Hill’s fitted model (lines).

Figure 4 shows the biodegradation kinetics in terms of percentage biodegradation (Equation (5)) as a function of time for the PLA-PHBV blend films, containing or not containing phenolic acids, in comparison with the CMC sample. In order to validate the quality of the inoculum used in the biodegradation test, ISO 14855 establishes that the degree of biodegradation of the reference material must be more than 70% after 45 days. As can be observed in Figure 4, the degree of biodegradation of the positive reference material (CMC) was 91% after 45 days of composting, which validates the experimental data.

All the PLA-PHBV blend films, containing or not containing phenolic acids, showed the characteristic sigmoid profiles of the respirometry test with the three different phases that were also found by other authors [37,39,40]: an initial delay/induction period (extended up to 10–22 days, depending on the type of film), followed by a biodegradation phase (extended up to 22–28 days) and a plateau. It is known that the degradation of PLA in compost takes place in two main, consecutive stages. The process starts at the surface level with the hydrolysis of the polymer chains induced by the diffusion of water from the materials, causing a random polymer decomposition to form oligomers and lactic acid. Subsequently, once the molecular weight reaches approximately 10,000–20,000 D, the enzymatic degradation takes place, leading to the formation of carbon dioxide, water and humus [41]. On the other hand, the degradation of PHBV is firstly caused by microorganisms through a mechanism of surface erosion, gradually spreading to the bulk [42]. Therefore, both water-induced hydrolysis in the polymer films and enzymatic degradation caused by microorganisms contributed to biodegradation [43], with abiotic hydrolysis being the main depolymerisation mechanism according to the higher ratio of PLA in the films. Therefore, any factor affecting the rate of hydrolysis could either accelerate or retard the whole degradation process [44].

Amorphous PLA-PHBV films presented an initial delay period of 10 days and completely biodegraded (B = 100%) after 20 composting days, in contrast to what was found by other authors for full crystalline PLA-PHBV films [31], which reached 90% biodegradation within 180 days. This difference can be explained by the amorphous nature of the PLA used in the film, as the crystalline regions of PLA are hydrolysis-resistant compared to the amorphous regions due to the highly restricted access of water molecules to chains inside the rigid crystalline regions [45]. Therefore, the use of amorphous PLA favours the hydrolytic cleavage of PLA chains, thus promoting a faster degradation pattern.

The addition of phenolic acids modified the biodegradation profile of PLA-PHBV films, especially when using protocatechuic acid. Thus, longer initial induction periods (lag phase) and a more active composting stage were detected for P-P films. The initial delay periods were of around 10–12 days for all the films, except for those containing protocatechuic acid, when this period was extended to 22 days. This could be linked to the different bonding capacity of the phenolic acids to the polymer chains that affects their release from the matrix and dilution in the compost medium. The debonding of the phenolic acids will also contribute to the hydrolysis process, given their low pK_a_ values (4.5–4.6) and capacity to acidify the aqueous environment. The release kinetics of these acids from the PLA-PHBV matrix into aqueous food simulants of differing polarities (controlled by the ethanol content) revealed that a greater amount of ferulic acid than *p*-coumaric was released and at a faster rate. The difference was even more marked if compared with protocatechuic acid, whose release was very limited [35]. The faster release of ferulic acid could have contributed to the acceleration of the hydrolyses/hydrolysis of the PLA amorphous matrix, shortening the induction time. In contrast, the antimicrobial capacity of these phenolic compounds and their bonding capacity to the polyester blend through hydrogen bonds with carboxylic oxygens [35] could also limit the accessibility of polymer matrices for an attack by microorganisms. This bonding capacity was greater in the case of protocatechuic acid, the only acid exhibiting two hydroxyl groups in the phenolic ring able to establish interchain hydrogen bonds with the polyester carbonyl groups.

PLA-PHBV blend films containing phenolic acids completely biodegraded after 20–27 days (B = 100%). Moreover, after 30 days, these films reached biodegradation percentages of over 100%. This effect is attributed to the priming effect brought about by the overstimulation of the organic matter mineralisation when using easily decomposable polymers, as was also found by other studies [46]. How the mechanism via the priming effect occurs is unknown [47], but it is believed to be the result of the interaction among the microorganisms and the small molecules released into the medium due to polymer degradation [29].

Phenolic acids exert antimicrobial activity by diffusion of the undissociated acid across the membrane, resulting in the acidification of the cytoplasm and, eventually, cell death [48,49,50,51]. There are several factors affecting this activity, such as the pK_a_ and the lipo-philicity of the phenolic acid (related to their molecular structure), since these determine the solubility of phenolic acids in bacterial membranes. Ferulic and *p*-coumaric acids (hydroxycinnamic acids) are more lipophilic than protocatechuic (which is a hydroxybenzoic acid) because of their side unsaturated chain and, thus, they could exhibit a greater antimicrobial activity. On the other hand, the methoxy group in ferulic acid makes this acid more lipophilic than *p*-coumaric [52], which could promote its antimicrobial activity in the inoculum. Nevertheless, apart from the different length of the induction period, the potential antimicrobial effect did not notably interfere in the biodegradation of the PLA-PHBV films, which reach total biodegradation during a relatively short composting time (20–27 days). Protocatechuic acid was the compound that promoted the longest delay, probably due to its greater persistency in the polymer matrix, as deduced in previous studies [35].

The biodegradation curves were fitted to the Hill equation, and the fitting parameters are reported in Table 4. The time required to reach 50% of the maximum biodegradation level (k values) in the blend films ranged from 17 to 26 days, with the longest time being for films with protocatechuic acid. The maximum biodegradation percentage (B_max_) ranged between 158 and 230, with the highest value corresponding to the PLA-PHBV blend films, while the values decreased in proportion to how difficult it is for the compound to gain release from the matrix deduced in previous studies [35]. The faster the release kinetics of the compound in aqueous media, the lower the B_max_ values, and the shorter the induction period of the biodegradation process. Therefore, although the presence of any of the studied phenolic acids does not inhibit the biodegradation process of the PLA-PHBV films, they tended to prolong the induction period, slightly reducing the percentage of final biodegradation when the compound was more tightly retained in the polymer matrix. This was probably due to there being greater difficulties for it to dilute in the media, making the antimicrobial action more effective. Similar results were obtained by other authors [37] analysing films loaded with non-volatile antimicrobial compounds.

## 4. Conclusions

This study provided relevant information regarding the effect that the incorporation of phenolic acids into PLA-PHBV blend films had on their disintegration and biodegradation processes. A complete disintegration of the PLA-PHBV blend films was observed during the 35 days of the composting test, regardless of the presence of phenolic acids. From the TGA, the faster disintegration of the amorphous PLA fraction could be deduced, while the process was barely affected by the presence of phenolic acids. As concerns the biodegradation process, the incorporation of the phenolic acids delayed the biodegradation period, extending the induction period, especially when using protocatechuic acid. This could be related to the greater persistency of the compound in the polymeric matrix. Phenolic-free PLA-PHBV blend films fully biodegraded after 20 composting days, while *p*-coumaric and protocatechuic slightly retarded full biodegradation (21 and 26 days, respectively). These results indicated that none of the antimicrobial phenolic acids inhibit the biodegradation process of the blend films, but they can retard biodegradation when these are tightly retained in the polymer matrix. Thus, these new films with potential antimicrobial activity could be used to preserve fresh foodstuff susceptible to microbial spoilage, with their biodegradation under composting conditions being ensured. Further studies should focus on biodegradation studies of these films exposed to different natural environments such as soil or sea water.

## Figures and Tables

**Table 1:** Moisture content (MC), thickness and elemental carbon (C%) of the films prior to the composting test. PLA-PHBV blend films (P) and PLA-PHBV blend films containing ferulic acid (P-F), *p*-coumaric acid (P-C) and protocatechuic acid (P-P). Mean values and standard deviations.

Sample/Reactor	MC (%)	Thickness (µm)	C (%)
P	0.1600 ± 0.0006 ^a^	146 ± 0.015 ^b^	52.0 ± 0.2 ^a^
P-F	0.1500 ± 0.0010 ^b^	153 ± 0.018 ^a^	52.0 ± 0.6 ^a^
P-C	0.0300 ± 0.0002 ^d^	144 ± 0.016 ^c^	52.1 ± 0.3 ^a^
P-P	0.1000 ± 0.0008 ^c^	137 ± 0.018 ^d^	52.0 ± 0.4 ^a^

Different superscript letters (a–d) within the same column indicate significant differences between formulations (*p* < 0.05).

**Table 2:** Volatile solids (VS) after the composting period of the disintegration test, the reduction in the volatile content (R) and disintegration percentage for the samples after 35 composting days of the SSR, control (P) and P films incorporating ferulic acid (P-F), *p*-coumaric acid (P-C) and protocatechuic acid (P-P). Mean values and standard deviations.

Reactor	VS (g Volatiles/100 g DS) Post-Composting	R (%)	Disintegration D_35_ (%)
SSR (blank)	90.5 ± 0.9 ^a^	40 ± 1.8 ^d^	-
P	86 ± 1.3 ^b^	58 ± 1.5 ^b^	89 ± 2 ^ab^
P-F	82 ± 1.0 ^c^	64 ± 1.6 ^a^	92 ± 5 ^a^
P-C	85 ± 1.2 ^b^	53 ± 0.6 ^c^	85 ± 3 ^b^
P-P	89 ± 1.6 ^a^	54 ± 2.0 ^c^	88 ± 4 ^b^

Different superscript letters (a–d) within the same column indicate significant differences between formulations (*p* < 0.05). (-) Reactor without sample.

**Table 3:** TGA parameters obtained from the TGA throughout the composting process: onset (To) and peak (Tp) temperatures, and pyrolysis residual mass at 600 °C of PLA-PHBV blend films (P) and PLA-PHBV blend films containing ferulic acid (P-F), *p*-coumaric acid (P-C) and protocatechuic acid (P-P). Mean values and standard deviation.

Sample	Day	T_o_	Tp_1_	Tp_2_	Residual Mass (%)
P	0	197.0 ± 3.0 ^c,1^	268.0 ± 3.0 ^b,1^	320.0 ± 3.0 ^a,1^	0.4 ± 0.1 ^b,2^
	14	181.0 ± 4.0 ^a,2^	210.0 ± 4.0 ^bc,2^	240.0 ± 4.0 ^c,2^	2.5 ± 0.5 ^b,1^
	21	180.8 ± 0.3 ^b,2^	207.4 ± 1.1 ^c,2^	-	4.0 ± 1.5 ^abc,1^
P-F	0	242.8 ± 2.0 ^a,1^	273.0 ± 1.0 ^a,1^	324.0 ± 3.0 ^a,1^	0.5 ± 0.1 ^ab,3^
	14	186.5 ± 0.1 ^b,2^	214.7 ± 0.2 ^b,2^	248.0 ± 0.7 ^ab,2^	1.1 ± 0.1 ^c,2^
	21	184.5 ± 1.0 ^a,3^	210.8 ± 2.0 ^b,3^	-	3.6 ± 0.2 ^b,1^
P-C	0	207.0 ± 3.0 ^b,1^	269.0 ± 1.0 ^b,1^	321.0 ± 1.0 ^a,1^	0.7 ± 0.1 ^a,2^
	14	184.9 ± 0.1 ^b,2^	212.9 ± 0.4 ^c,2^	247.3 ± 1.0 ^b,2^	1.9 ± 0.1 ^b,1^
	21	184.2 ± 0.5 ^a,2^	211.8 ± 0.5 ^a,3^	-	2.3 ± 0.5 ^c,1^
P-P	0	176.0 ± 2.0 ^d,3^	232.0 ± 3.0 ^c,1^	275.0 ± 1.0 ^b,1^	0.6 ± 0.1 ^ab,3^
	14	186.9 ± 0.2 ^b,1^	215.2 ± 0.1 ^a,2^	249.1 ± 0.5 ^a,2^	3.8 ± 0.6 ^a,2^
	21	183.6 ± 1.0 ^a,2^	210.6 ± 0.1 ^b,3^	-	5.0 ± 0.5 ^a,1^

Different superscript letters (a–d) within the same column indicate significant differences between formulations after the same analysis time (*p* < 0.05). Different superscript numbers (1–3) within the same column indicate significant differences between the composting times for a given formulation (*p* < 0.05).

**Table 4:** Hill’s parameters (%B_max_, k and n) for the different films and cellulose microcrystalline (CMC, reference). k: time needed to reach 50% of B_max_, and B_max_: percentage of biodegradation at infinite time.

Sample	n	k (days)	B_max_ (%)	R^2^
CMC	1.8	15.5	105	0.92
P	8.0	19.5	226	0.89
P-F	10.1	17.1	200	0.80
P-C	9.0	20.3	170	0.83
P-P	39.1	25.6	156	0.78

n: curve radius of the sigmoid function; k: time required to reach 50% of the maximum biodegradation; B_max_ (%): Percentage of biodegradation at infinite time; R^2^: correlation coefficient.
